# Downstream processing of recombinant human insulin and its analogues production from *E. coli* inclusion bodies

**DOI:** 10.1186/s40643-021-00419-w

**Published:** 2021-07-27

**Authors:** Yin Yin Siew, Wei Zhang

**Affiliations:** Downstream Processing Group, Bioprocessing Technology Institute, Agency for Science, Technology and Research, Singapore, Singapore

**Keywords:** Recombinant human insulin, Insulin analogues, *E. coli* inclusion bodies, Downstream processing, Purification

## Abstract

The Global Diabetes Compact was launched by the World Health Organization in April 2021 with one of its important goals to increase the accessibility and affordability of life-saving medicine—insulin. The rising prevalence of diabetes worldwide is bound to escalate the demand for recombinant insulin therapeutics, and currently, the majority of recombinant insulin therapeutics are produced from *E. coli* inclusion bodies. Here, a comprehensive review of downstream processing of recombinant human insulin/analogue production from *E. coli* inclusion bodies is presented*.* All the critical aspects of downstream processing, starting from proinsulin recovery from inclusion bodies, inclusion body washing, inclusion body solubilization and oxidative sulfitolysis, cyanogen bromide cleavage, buffer exchange, purification by chromatography, pH precipitation and zinc crystallization methods, proinsulin refolding, enzymatic cleavage, and formulation, are explained in this review. Pertinent examples are summarized and the practical aspects of integrating every procedure into a multimodal purification scheme are critically discussed. In the face of increasing global demand for insulin product, there is a pressing need to develop a more efficient and economical production process. The information presented would be insightful to all the manufacturers and stakeholders for the production of human insulins, insulin analogues or biosimilars, as they strive to make further progresses in therapeutic recombinant insulin development and production.

## Introduction

Diabetes is a global epidemic (WHO 2021). In April 2021, the World Health Organisation has launched the Global Diabetes Compact to hasten action to tackle diabetes worldwide. More than 420 million (6% of the world’s population) were living with diabetes before COVID-19 emerged, and it is predicted that this number would surge to 500 million by 2030, and to 700 million by 2045 (WHO 2021). The rise in the number of diabetics worldwide would lead to the consequential increase in the demand for insulin. In addition, there is an unmet demand for affordable insulin, especially in low- and middle-income nations. Many people with type 1 diabetes who are completely reliant on insulin for survival have no access to insulin (WHO 2021). For the 60 million people with type 2 diabetes who need insulin treatment, 1 in 2 of them do not get insulin due to its price (WHO 2021). In 2018, the world human insulin market size was USD 21.26 billion and this number is expected to reach USD 27.71 billion by 2026 (Fortune Business Insights 2020). The human insulin market is forecasted to expand at a compound annual growth rate (CAGR) of 3.4% from 2019 to 2026. In terms of human insulin market dominance, three major companies, Sanofi, Novo Nordisk A/S and Elli Lily and Company, are collectively holding more than 90% share of the market revenue (Fortune Business Insights 2020).

Injections of insulin are important to treat both type 1 and type 2 diabetes, and recombinant human insulin has been shown to have significant advantages over insulins extracted from pork and beef sources. In 1978, scientists at City of Hope first successfully produced recombinant human insulin in partnership with Genentech (Chance and Frank 1993). Eli Lilly and Company launched human insulin of recombinant DNA origin in 1982, while Novo launched it in 1988. Two different production approaches have been reported from Eli Lilly and Company. In the first approach, the A and B chains of insulin were cultured separately in bacteria as inclusion bodies. Then, separate purifications of the two chains were performed and later combined chemically, followed by eventual steps of purification. In the second technique, the expression of proinsulin in *Escherichia coli* took the form of a tryptophan promotor with a methionine linkage to proinsulin. Cyanogen bromide (CNBr) was used to cleave the linkage, followed by folding of the peptide via correct disulfide bonds formation, and the eventual elimination of the C-peptide via enzymatic action (Frank et al. 1981). It is preferable to adopt the “proinsulin route” as it only requires a single fermentation and purification procedure, which makes the production process more efficient in comparison to the two-chain combination method. Since 1986, the “proinsulin route” has been adopted commercially (Chance et al. 1999). Eli Lilly used this technology in partnership with Genentech to produce Humulin, the first of several recombinant insulins to be approved for general medical use (Walsh 2005).

Besides using bacteria as the host system, yeast has also been included in the commercial production of insulin. The predominant yeast strains used commercially are *Saccharomyces cerevisiae* and *Pichia pastoris.* Yeast-based expression system yields soluble insulin precursors which are secreted into the culture supernatant. There are different pros and cons to using bacteria and yeast as expression systems. Production of precursor insulin via the bacterial inclusion body route typically yields higher product concentration and productivity (Baeshen et al. 2014). Compared to soluble expressions of bioactive therapeutic proteins, the inclusion bodies have good mechanical stability and are resistant to proteolytic degradation (Singhvi et al. 2020). In terms of downstream purification, the protein of interest from inclusion bodies is also isolated in a purer and more concentrated state compared to secreted proteins which require the removal of abundant host cell protein impurities from the culture supernatant. The isolation of peptide from inclusion bodies also tends to be easier due to the differences in their size and density as compared with host proteins (Singhvi et al. 2020).

Using *E. coli* as the expression system for large-scale recombinant insulin production possesses the advantages of high growth rate, simple media requirement, ease of handling, high yield, and cost effectiveness (Baeshen et al. 2014). *E. coli* also has well-characterized genetics, and an availability of a huge quantity of mutant host strains and cloning vectors (Baneyx 1999). Nevertheless, the inclusion body route of production from *E. coli* requires complicated and extensive processing, such as solubilization and refolding procedures, to obtain fully functional polypeptides. Table 1 provides a list of insulin products currently on the market and their host systems. For most of the biopharmaceutical companies, *E. coli* remains the host system of choice to produce recombinant insulin, with human insulin and its analogues expressed in inclusion bodies in most instances. Insulin analogues are synthetically produced variations of insulin that have a different amino acid sequence to native human insulin (Chouhan et al. 2017). Such alterations have been made to more closely mimic the normal physiologic pattern of insulin secretion in the human body (Freeman 2009).

**Table 1:** Commercial recombinant human insulin/insulin analogue products and their production host systems

Insulin type	Structure	Action	Host system	Manufacturer	Brands
Human insulin	Identical to native human insulin	Fast/short/intermediate/long-acting depending on formulation	*E. coli*	Berlin-Chemie	Berlinsulin
				Bioton	Gensulin
				Eli Lilly & Co	Huminsulin, Humulin
				Landsteiner Scientific	Bonglixan
				Sanofi	Insulin Human Winthrop, Insuman
				SciGen Ltd	Scilin
				Tonghua Dongbao	Gansulin
			*H. polymorpha*	Wockhardt	Wosulin
			*P. pastoris*	Biocon	Insugen
			*S. cerevisiae*	Novo Nordisk	Actraphane, Actrapid, Insulatard, Mixtard, Monotard, Novolin, Protaphane, Ultratard, Velosulin
		Inhalable; Ultra rapid-acting	*E. coli*	MannKind	Afrezza
Insulin lispro	Engineered: inversion of native B28–B29 proline-lysine sequence	Fast-acting	*E. coli*	Eli Lilly & Co	Humalog, Liprolog
				Sanofi	Admelog
		Short-acting	*E. coli*	Gan & Lee	Prandilin
Insulin glargine	Engineered: A 21 asparagine replaced by glycine and B chain elongated by two arginines	Long-acting	*E. coli*	ACI Limited	Glarine
				Eli Lilly & Co	Abasaglar, Basaglar
				Gan & Lee	Basalin
				Getz Pharma	Basagine
				Incepta Pharmaceuticals	Vibrenta
				Merck	Lusduna Nexvue
				Sanofi	Lantus, Optisulin, Toujeo
				Wockhardt	Glaritus
			*P. pastoris*	Biocon	Basalog
Insulin aspart	Engineered: B28 proline replaced by aspartic acid	Fast-acting	*S. cerevisiae*	Novo Nordisk	NovoRapid, Novolog, Fiasp
Insulin glulisine	Engineered: B3 asparagine is replaced by a lysine and B29 lysine is replaced by glutamic acid	Fast-acting	*E. coli*	Sanofi	Apidra
Insulin detemir	Engineered: devoid of B30 threonine and a C14 fatty acid is covalently attached to B29 lysine	Long-acting	*S. cerevisiae*	Novo Nordisk	Levemir
Insulin degludec	Engineered: devoid of B30 threonine and hexadecanedioic acid via gamma-l-glutamyl spacer is conjugated to B29 lysine	Ultra-long acting	*S. cerevisiae*	Novo Nordisk	Tresiba

**Fig. 1:**
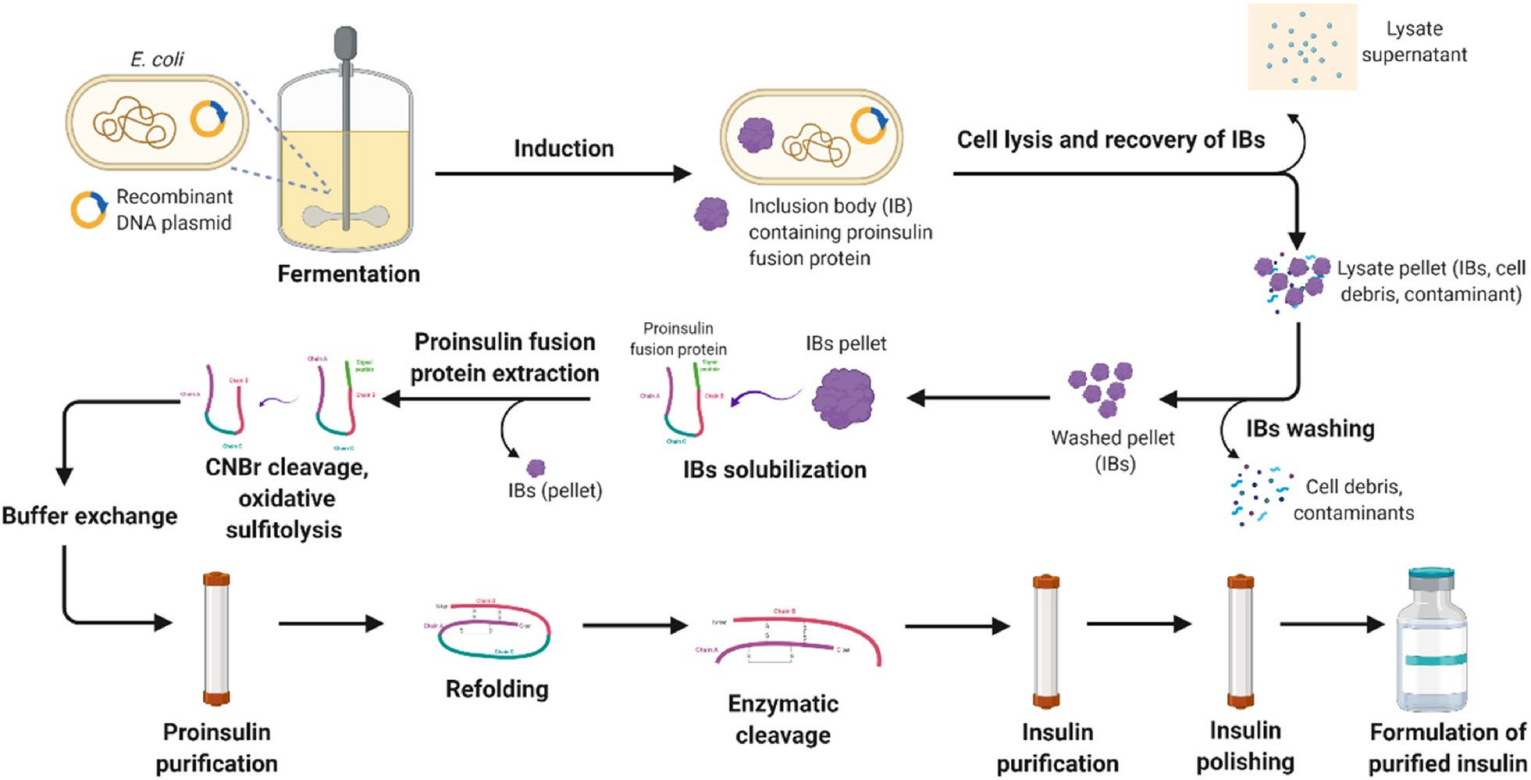
The general workflow for the downstream processing of recombinant human insulin and its analogues (created with BioRender.com)

The single-chain insulin precursor molecules sequestered in *E. coli* inclusion bodies are mostly misfolded. The inclusion bodies are extracted from the cells, washed, and the precursor molecules are then solubilized. Production of proinsulin requires both peptide folding and concomitant formation of disulfide bonds. After enzymatic reaction, the refolded precursor molecule is then converted into a heterodimer insulin molecule by the removal of the C-chain and the N-terminus fusion peptide. Various chromatographic purification steps are required to remove host cell proteins, nucleic acids, cell membrane fragments, and digestion by-products, to yield a highly purified product. The general workflow for the downstream processing of recombinant human insulin and its analogues is summarized in Fig. 1.

The downstream process to purify recombinant human insulin/analogues from *E. coli* inclusion bodies is complex and involves multiple steps, and the specifics of the processes established by different companies are proprietary. The approaches adopted in each step will have a big impact on the next step in terms of product purity and yield. Here, we present a comprehensive review of downstream processing of recombinant human insulin/analogues production from *E. coli* inclusion bodies*.* All the critical aspects of downstream processing of recombinant human insulin/analogues produced in *E. coli* inclusion bodies will be summarized and discussed.

### Recovery of inclusion bodies

The heterologous proteins are expressed in the form of insoluble cytoplasmic inclusion bodies and they are not being excreted into the culture media. Therefore, the inclusion bodies have to be recovered from the bacterial cells by either a mechanical or lysozyme-based method (Astolfi Filho et al. 2004; Singh et al. 2015). Mechanical methods to rupture the cell envelope involve using sonication, grinding the cell suspension in a colloidal mill such as a Dyno-Mill, or by passing the cell paste through a Manton-Gaulin press or French press. In a lysozyme-based method, the lysozyme is added to digest the cell envelope. Thereafter, the inclusion bodies, which are denser than the other cellular components, can be easily isolated from the whole cell lysate by techniques such as membrane microfiltration or centrifugation. Generally, mechanical methods can effectively disrupt cells, but may compromise the protein quality in inclusion bodies more than lysozyme-based methods (Singhvi et al. 2020). Neither centrifugation nor membrane filtration has any effect in the extraction and washing of inclusion bodies (Tikhonov et al. 2001).

### Inclusion body washing

Inclusion bodies recovered from bacterial cell lysates can be heavily contaminated with intact whole cells, host proteins, RNA, peptidoglycan cell wall, and membrane fragments (Schoner et al. 1985; Singh et al. 2015). After lysis of the *E. coli* cells, the inclusion bodies have to be washed sequentially to remove contaminants which often have strong ionic and hydrophobic interactions with the inclusion body proteins (Astolfi Filho et al. 2004). Inclusion body washing is critical in recombinant insulin purification, without which numerous impurities will persist and may interfere with the following steps, such as sulfitolysis, renaturation, and enzymatic digestion (Min et al. 2011). This could lead to a reduction in purification yield.

Recovery of washed inclusion bodies is performed by multiple rounds of centrifugation combined with re-suspension and washing of pellets with detergents and denaturants. After the wash, the supernatant is discarded to leave behind the inclusion body pellet. Alternatively, instead of using centrifugation, isolation of inclusion bodies from bacteria can also be achieved via membrane filtration (Yuan et al. 2015).

A list of additives used in washing the proinsulin fusion protein-containing inclusion bodies are summarized in Table 2. The two most common wash buffer additives are urea and Triton X-100. The *E. coli* cell wall is constituted of phospholipid, protein, peptidoglycan, and lipopolysaccharide (Clark 1998). Selective extraction with detergents, low concentrations of urea, lysozyme, and EDTA facilitates the removal of these bacterial cell wall components. Typically, the pH of the wash buffers ranged from pH 7.4 to pH 8.0. Wash optimization is required to evaluate the efficacy of additives in removing impurities. It is also important to be mindful of ensuring the wash buffer does not inadvertently solubilize the desired recombinant protein, which may negatively impact its recovery. Other time and cost-saving optimization include number of centrifugation rounds, speed of centrifugation, and wash temperature.

**Table 2:** A list of additives, their specific functions, and pertinent examples of proinsulin fusion protein-containing inclusion body washes

Additives	Functions	Concentrations	Example references
EDTA	A metal chelator to inactivate metalloproteases (Ritchie 2012) Disrupts the lipopolysaccharide site of insertion into the bacterial outer membrane (Schnaitman 1971)	0.5 mM	Mackin (2014)
		1 mM	Chen et al. (2016) Hwang et al. (2016) Nilsson et al. (1996) Yuan et al. (2015)
		2 mM	Leng et al. (2013) Tikhonov et al. (2001)
Glycerol	NA	5%	Mackin (2014)
Lysozyme	Cleaves the backbone of peptidoglycan (Hwang et al. 2016)	0.02%	Hwang et al. (2016) Kim et al. (2015) Son et al. (2009)
NaCl	Solubilizes impurity through ionic interaction (Ritchie 2012)	0.05 M	Mackin (2014)
		0.1 M	Leng et al. (2013)
		0.2 M	Nilsson et al. (1996)
		0.5 M	Mikiewicz et al. (2017) Yuan et al. (2015) Zieliński et al. (2019)
		NA	Zimmerman and Stokell (2010)
Triton X-100	Removes membrane phospholipid and fragments (Singh et al. 2015; Palmer and Wingfield 2012) Facilitates dissociation of debris and soluble proteins from inclusion bodies (Petrides et al. 1995)	0.66%	Petrides et al. (1995)
		1%	Chen et al. (2016) Hwang et al. (2016) Kim et al. (2015) Leng et al. (2013) Mikiewicz et al. (2017) Min et al. (2011) Son et al. (2009) Tikhonov et al. (2001) Zieliński et al. (2019)
		2%	Castellanos-Serra et al. (1996) Cowley and Mackin (1997)
		NA	Zimmerman and Stokell (2010)
Tween 20	Extracts the bacterial outer membrane components (Palmer and Wingfield 2012)	0.05%	Nilsson et al. (1996) Zimmerman and Stokell (2010)
		NA	Zimmerman and Stokell (2010)
Urea	A chaotropic agent to extract the bacterial outer membrane components (Palmer and Wingfield 2012; Petrides et al. 1995)	0.5 M	Hwang et al. (2016) Kim et al. (2015)
		2 M	Chen et al. (2016) Cowley and Mackin (1997) Kim et al. (2015) Min et al. (2011) Son et al. (2009) Yuan et al. (2015)
		3 M	Tikhonov et al. (2001)
		4 M	Castellanos-Serra et al. (1996)

*NA* information not available

### Inclusion body solubilization and oxidative sulfitolysis

Inclusion bodies contain protein in a stable non-native conformation. The protein aggregates may be amorphous, with partial or complete denaturation (Astolfi Filho et al. 2004). Inclusion bodies are relatively insoluble in aqueous buffers and this has introduced substantial challenges in purification. Therefore, the washed inclusion bodies have to be solubilized with solubilization buffer solution to recover the recombinant protein.

Inclusion bodies are conventionally solubilized using high concentration of denaturants, such as guanidine hydrochloride (GdnHCl) and urea, which results in a complete disruption of protein structure (Singh et al. 2015). For proteins that contain numerous cysteine residues, dithiothreitol (DTT) or β-mercaptoethanol (BME) may be added to reduce incorrect disulfide-bond formation (Harrison et al. 2015). Triton X-100 and sodium dodecyl sulfate can be used to extract proteins from inclusion bodies (Astolfi Filho et al. 2004), though their usage is rather uncommon in proinsulin fusion protein solubilization.

Oxidative sulfitolysis involves the addition of –SO_3_ groups to the –SH of cysteine residues in proinsulin polypeptides to form protein-*S*-sulfonate in the presence of a high concentration of denaturing reagent (e.g., 6 M GdnHCl or 8 M urea) (Frank et al. 1981; Petrides et al. 1995). As proinsulin expressed in *E. coli* is not folded in the correct conformation, the sulfitolysis step is crucial to maintain its unfolded form (Petrides et al. 1995). This will prevent the formation of potentially incorrect disulfide bonds in solubilization and during initial purification steps preceding protein renaturation (Harrison et al. 2015). Furthermore, oxidative sulfitolysis can aid in improving the subsequent refolding yield (Min et al. 2011).

Some workflows would start with inclusion body solubilization first, followed by oxidative sulfitolysis (Min et al. 2011; Petrides 1995). The conditions for solubilization of proinsulin fusion protein-containing inclusion bodies (without concurrent oxidative sulfitolysis), ranked in order of ascending duration of solubilization, are presented in Table 3. The 8 M urea is the most common solubilization agent. Reducing agents (e.g., BME, DTT) are added to break the disulfide bonds. Table 4 presents examples in which inclusion body solubilization was carried out simultaneously with oxidative sulfitolysis in human insulin/analogue production, and these conditions are listed and ranked in order of ascending duration of reaction. Sulfitolysis of denatured proinsulin is carried out with sodium sulfite and sodium tetrathionate at a molar ratio of at least 2:1 (Table 4). In oxidative sulfitolysis, sodium sulfite assumes the role as a reducing agent. Sodium tetrathionate, as an oxidizing agent, is preferred over cystine, Ni^2+^ and Cu^2+^ ions, as the former requires a shorter reaction time to reach maximum yield (Min et al. 2011; Tikhonov et al. 2001). To achieve maximal reaction effectiveness, it is critical to use only freshly prepared tetrathionate (Tikhonov et al. 2001). If not, the formation of side products could affect the yields and subsequent folding step.

**Table 3:** Conditions for solubilization of inclusion bodies reported in human insulin/analogue downstream processing, ranked in order of ascending duration of solubilization

Solubilization duration	Solubilization agent	Reducing agent	Chelating agent (EDTA)	Other components	Temp. (°C)	pH	Suspension concentration during solubilization (mg/mL)	References
30 min	8 M urea	–	–	–	NA	8.9	NA	Castellanos-Serra et al. (1996)
30 min	–	–	0.2 mM	12 mM NaHCO_3_	RTP	12.0	NA	Mikiewicz et al. (2017)
45 min	–	–	0.2 mM	12 mM NaHCO_3_	RTP	11.9, then adjusted to 10.8	NA	Zieliński et al. (2019)
6 h	8 M urea	–	5 mM (optional)	1% toluene (optional)	RTP	8.5	NA	Astolfi et al. (2004)
8 h	5 M urea	40 g/L BME	–	–	NA	NA	NA	Petrides (1995)
Overnight	8 M urea	3 mM BME	1 mM	–	4	8.0	100	Bai et al. (2003)
> 12 h	8 M urea	20 mM DTT	1 mM	–	4	8.0	100	Yuan et al. (2015)
NA	6 M GdnHCl	100 mM BME	–	–	NA	8.5	167	Chen et al. (2016)
NA	4 M urea	–	–	10 mM glycine	NA	10.6	NA	Son et al. (2009)
NA	8 M urea	–	–	–	NA	10.6	NA	Hwang et al. (2016)
NA	8 M urea	–	1 mM	–	NA	11, then adjusted to 9.5	10–15	Kim et al. (2015)
NA	8 M urea	4 mM BME	–	–	NA	10.4	20–30	Leng et al. (2013)
NA	8 M urea	–	1 mM	–	NA	11, then adjusted to 9.5	10–15	Min et al. (2011)
NA	8 M urea and 6 M GdnHCl	–	–	–	NA	9.0	10	Redwan et al. (2007)
NA	8 M GdnHCl	–	–	–	NA	10.8	NA	Thurow et al. (2010)

*NA* information not available, *RTP* room temperature

**Table 4:** Oxidative sulfitolysis conditions reported in human insulin/analogue downstream processing, ranked in order of ascending duration of reaction

Duration of oxidative sulfitolysis	Temp	pH	Sodium sulfite (A)	Sodium tetrathionate (B)	Molar ratio of (A): (B)	Solubilization agent	Suspension concentration during oxidative sulfitolysis (mg/mL)	References
40 min	37 °C	8.7	0.4 M	0.15 M	2.6:1	7.5 M urea	15–20	Tikhonov et al. (2001)
1–2 h	RTP	8.5–8.7	0.1 M	0.01 M^a^	10:1	7 M GdnHCl	NA	Patrick and Lagu (1992)
2–3 h	RTP	8.7	0.4 M	0.15 M	2.6:1	7.5 M urea	15–20	Tikhonov et al. (2001)
3 h	RTP	8.2	0.10 M	0.01 M	10:1	7 M urea	50	Cowley and Mackin (1997)
4 h	25 °C	9.5	0.2 M	0.02 M	10:1	8 M urea	10–15	Kim et al. (2015)
4 h	25 °C	11	0.2 M	0.02 M	10:1	8 M urea	10–15	Min et al. (2011)
6 h	NA	8.9	NA	NA	2:1	8 M urea	3	Castellanos-Serra et al. (1996)
6 h	37 °C	NA	0.8 M	0.3 M	2.6:1	8 M urea	2	Nilsson et al. (1996)
12 h	4 °C	9.5	0.2 M	0.02 M	10:1	8 M urea	10–15	Min et al. (2011)
12 h	NA	9–11	3% w/w	1.5% w/w	4.9:1	6 M GdnHCl	NA	Petrides et al. (1995)
12 h (24 h for pretreated cells)	RTP	NA	0.4 M	–^b^	–	8 M urea and 6 M GdnHCl	10	Redwan et al. (2007)
24–48 h	RTP	9.0	1.25 g/g of sample	0.55 g/g of sample	5.5:1	8 M urea	NA	Astolfi et al. (2004)

*NA* information not available, *RTP* room temperature

^a^Potassium tetrathionate was used

^b^0.4 mM cystine, 1 mM copper sulfate pentahydrate, and 5 mM nickel (II) chloride hydrate were used

pH and temperature are factors which could affect the solubilization and sulfitolysis rate of reaction. The solubilization and sulfitolysis reaction are typically carried out under alkaline conditions above pH 8 (Tables 3, 4). Min et al. (2011) and Kim et al. (2015) have reported using pH adjustment to 11 then to 9.5 to achieve higher protein solubility. The pH adjustment to 10.5 could attain complete protein solubilization (Zimmerman and Stokell 2010). Raising both the pH (from pH 9.5 to 11) and temperature (from 4 to 25 °C) of the sulfitolysis buffer will shorten the reaction time from 12 to 3 h (Min et al. 2011). The sulfitolysis reaction can be completed in 40 min at 37 °C, as compared to 2–3 h at room temperature (Tikhonov et al. 2001).

To achieve maximum yield, it is important to monitor the temperature and duration of the sulfitolysis reaction to ensure that the reaction goes to completion. However, going beyond what is required could be undesirable for the following fusion protein isolation and folding steps as the harsh conditions could lead to irreversible changes in protein structure (Tikhonov et al. 2001). Analysis of fraction aliquots on a Mono-Q column can be performed to monitor the progression of sulfitolysis reaction (Astolfi et al. 2004). A straightforward way to stop the reaction is by diluting the sample with water (Castellanos-Serra et al. 1996; Kim et al. 2015; Min et al. 2011; Nilsson et al. 1996) or freezing on dry ice (Cowley and Mackin 1997). Buffer exchange and desalting can be carried out to remove the sulfitolysis salts (see "Buffer exchange" section).

### Cyanogen bromide (CNBr) cleavage

In *E. coli* protein translation, the recombinant protein must be translated as a fusion protein in which the N-terminal extension provides the initiator *N*-formylmethionine (fMet) (Laursen et al. 2005). fMet is a modified methionine used as the first amino acid in most bacterial proteins. Since fMet is recognized by the human immune system as a foreign body, it is important to remove it so as to avoid unwanted immunogenic reaction. To remove the signal sequence, the methionine linker of proinsulin can be cleaved off with cyanogen bromide (CNBr) before purification. In short, this process involves the suspension of protein sample in 70% formic acid and incubation with CNBr at room temperature in darkness for 12–16 h (Cowley and Mackin 1997; Mackin and Choquette 2003; Petrides et al. 1995). The disadvantages of this method are low cleavage specificity, prolonged evaporation of CNBr, high toxicity and volatility of CNBr, and possible chemical modifications of the released products (Mackin and Choquette 2003). An alternative method uses protein proteases to cleave the fusion proteins, though one has to be cautious to ensure that the protease cleaves at the correct site to remove N-terminal fused peptide.

### Buffer exchange

Some of the reagents used in protein extraction, sample preparation, and purification may have adverse effects on protein function and stability. This could interfere with subsequent downstream processes. Therefore, it is necessary to remove or reduce these contaminants using one or more protein clean-up methods. The aim is to make the extracted or purified protein samples compatible with subsequent downstream applications. Here, we discuss the usage of buffer exchange techniques (i.e., dialysis, diafiltration, and size exclusion chromatography) in human insulin/analogue production.

#### Dialysis

Dialysis is the traditional method for desalting or buffer exchange, using osmotic pressure to drive solutes across a membrane (Merck Millipore 2021). The main disadvantages of dialysis are extended time taken to complete the exchange and the requirement of a large surface membrane area for exchange. It is however suitable for sensitive proteins which precipitate easily. In laboratory scale, the solution containing solubilized proinsulin-containing inclusion bodies can be dialyzed to eliminate urea (Nilsson et al. 1996).

#### Diafiltration

Diafiltration achieves desalting or buffer exchange through the use of centrifugal force or other external pressure to drive small microsolutes through a porous membrane (Merck Millipore 2021). The membrane does not allow macrosolutes bigger than the pore size to pass through. The main advantage of diafiltration lies in its ability to concentrate protein samples.

In the industry, diafiltration is applied after major reaction steps to remove interfering reagents. For example, diafiltration is applied after inclusion body solubilization and sulfitolysis reaction to remove the high concentration of urea and sulfitolysis reagents (Petrides et al. 1995). Besides that, the renatured samples can be concentrated and buffer exchanged by diafiltration into a suitable buffer for enzymatic conversion to occur (Astolfi et al. 2004; Nilsson et al. 1996; Petrides et al. 1995; Zimmerman and Stokell 2010). A recovery yield as high as 95%–98% has been reported with diafiltration (Petrides et al. 1995).

#### Size exclusion chromatography (SEC)

The third method for desalting and buffer exchange is to use size exclusion chromatography (SEC), also known as gel filtration chromatography. SEC separates molecules according to their relative sizes (Pharmacia Biotech 2021). Using a group separation technique, the small molecules, such as salts, can be separated from the larger peptides. SEC is a faster alternative to dialysis, and it requires a low dilution factor. For human insulin/analogue production, Sephadex G-25 resin has been widely used in desalting. It is useful for the removal of salts and other small impurities from molecules with molecular mass above 5000 Da (Cytiva 2021). The use of Sephadex G-25 for buffer exchange has been reported after sulfitolysis reaction (Cowley and Mackin 1997) and before proinsulin renaturation (Astolfi et al. 2004; Cowley and Mackin 1997). There was also a report using Sephadex G-25 after lysis, but the gel filtration media became heavily contaminated with non-chromatographable material that they were discarded after every experiment (Mackin 1999). It is also possible to use Sephadex G-25 after the RP-HPLC polishing step to exchange the sample buffer and to remove residual acetonitrile (Zieliński et al. 2019). Bio-Gel P2 was used to remove formic acid and remaining cyanogen bromide after fusion protein cleavage (Mackin and Choquette 2003).

### Purification by chromatography and precipitation

Table 5 provides a summary of reported downstream purification schemes for recombinant human insulin/analogues produced in *E. coli* inclusion bodies. The entire purification process is based on a combination of different modes of chromatography which exploits differences in size, molecular charge, and hydrophobicity. After recovering fusion precursor peptide from inclusion body, the following capture step usually entails affinity chromatography (AC) or ion-exchange chromatography (IEX). It is important to ensure that the intermediate product is of high purity prior to enzymatic conversion step. IEX is also the chromatography of choice following protein renaturation and enzymatic conversion step. For insulin polishing step, it is common to use reversed-phase chromatography (RP) and size exclusion chromatography (SEC). Some of the purification strategies have incorporated folding and cleavage step early in the scheme, thus completely eliminating the need to purify insulin precursor. The renaturation of insulin precursor immediately following their recovery from inclusion body also eliminates the need to work with a high concentration of urea.

**Table 5:** A summary of reported downstream purification schemes for recombinant human insulin/analogues and their precursors produced in *E. coli*

Fusion protein expressed in *E. coli*		Downstream purification schemes (purification steps arranged in sequence)							References
rhPI	***IB recovery***	*IMAC*	*⭮*	*✁*	IEX	RP			Astolfi et al. (2004)
rhPI	***IB recovery***	*HIC (+ ⭮)*							Bai et al. (2003)
rhPI	***IB recovery***	*pH-PPT*	*AEX*	*pH-PPT*	*⭮*	*RP*	*✁*	RP	Castellanos-Serra et al. (1996)
rhPI	***IB recovery***	*AEX*	*⭮*	*RP*					Cowley and Mackin (1997)
rhPI	***IB recovery***	*MMC*	*Cryst*	*⭮*	*✁*	CEX			GE app notes 28-9966-22 AA (2012), 29-0018-56 AB (2012), Heldin et al. (2014)
rhPI	***IB recovery***	*pH-PPT*	*⭮*	*pH-PPT*	*✁*				Kim et al. (2015)
rhPI		*✁*	IEX	RP	SEC				Kroeff et al. (1989)
rhPI	***IB recovery***	*AC*	*⭮*	*RP*					Mackin (1999)
rhPI	***IB recovery***	*⭮*	*RP*	*✁*					Mackin and Choquette (2003)
rhPI	***IB recovery***	*pH-PPT*	*⭮*	*pH-PPT*	*✁*				Min et al. (2011)
rhPI	***IB recovery***	*⭮*	*AC*	*✁*	RP				Nilsson et al. (1996)
rhPI	***IB recovery***	*CEX*	*⭮*	*HIC*	*✁*	IEX	RP	SEC	Petrides et al. (1995)
rhPI	***IB recovery***	*IMAC*	*ZnCl*_*2*_*-PPT*	*CEX*	*⭮*	*✁*			Redwan et al. (2007)
rhPI	***IB recovery***	*IMAC*							Yuan et al. (2015)
rhPI	***IB recovery***	*SEC (+ ⭮)*							Yuan et al. (2015)
rhPI	***IB recovery***	*⭮*	*✁*	AEX	ZnCl_2_-PPT	AEX	✁	RP	Zieliński et al. (2019)
rhPI, analogues		*✁*	CEX						Coleman et al. (2019)
rhPI, analogues	***IB recovery***	*⭮*	*AEX*	*CEX*					Thurow et al. (2010)
rhPI, analogues	***IB recovery***	*AEX*	*IMAC*						Tikhonov et al. (2001)
rhPI, analogues	***IB recovery***	*IMAC*	*AEX*						Tikhonov et al. (2001)
rhPI, analogues		*✁*	MMC	RP					Watson et al. (2018)
rhPI, analogues	***IB recovery***	*⭮*	*✁*	AEX	AEX	✁	RP		Mikiewicz et al. (2017)
PI analogues	***IB recovery***	*⭮*	*IMAC*	*✁*	RP				Zimmerman and Stokell (2010)
PI aspart	***IB recovery***	*⭮*	*AEX*						Chen et al. (2016)
PI glargine	***IB recovery***	*⭮*	*pH-PPT*	*✁*	CEX	RP			Hwang et al. (2016)

Molecule		
***Inclusion body (IB)***	*Precursor of human insulin/insulin analogue *	Human insulin/insulin analogue

Chromatography		
AC: affinity chromatography	HIC: hydrophobic interaction chromatography	MMC: mixed-mode chromatography
AEX: anion exchange chromatography	IEX: ion-exchange chromatography	RP: reversed-phase chromatography
CEX: cation exchange chromatography	IMAC: immobilized metal ion affinity chromatography	SEC: size exclusion chromatography

Inclusion body (IB) (represented in bold italics format)

Precursor of human insulin/insulin analogue (represented in italics format)

Human insulin/insulin analogue (represented in underlined format)

*Cryst.* crystallization, *IB recovery* protein recovery from inclusion bodies, *pH-PPT* pH precipitation, *ZnCl*_*2*_*-PPT* zinc chloride precipitation, *rhPI* Recombinant human proinsulin

⭮: Renaturation

(+ ⭮): Simultaneous purification and renaturation

✁: Enzymatic conversion (e.g., Citraconylation & Trypsinization, Cleavage by trypsin and/or CPB)

Some of the more unconventional techniques in insulin downstream processing have been explored. The use of simulated moving bed (SMB), in place of traditional batch chromatography mode, has been reported for the purification of insulin (Wang et al. 2003). The SMB mode uses solid phase much more efficiently and require much less column volume for the same throughput. Furthermore, it can produce products at a purity similar to or higher than that of batch chromatography, and at substantially higher yields. There have been investigations on the use of protein-folding liquid chromatography (PFLC) to achieve simultaneous purification and renaturation of proinsulin (Bai et al. 2003; Yuan et al. 2015). A proof-of-concept using sub/supercritical fluid chromatography (SFC) to replace RP in insulin purification has been proposed (Govender et al. 2020). The SFC is more ecologically friendly as the major mobile phase, carbon dioxide, can be efficiently recycled. One of the SFC columns tested, the pentafluorophenyl column, was shown to yield a recovery of 84% in contrast to conventional RP-HPLC methods of ≥ 75%.

#### Affinity chromatography (AC)

AC is a separation technique based on specific binding interaction between an immobilized ligand and its binding partner. Depending on the specificity of the interaction, the degree of purification can be quite high. Some of the reported usage of AC in proinsulin purification is presented in Table 6. For AC to work, a suitable fusion tag has to be fused to the proinsulin molecule. Table 7 provides a list of fusion tags adopted in various insulin purification schemes and their advantages. Polyhistidine tag is the most commonly adopted fusion tag in insulin production. For affinity capture of His-tagged proinsulin, it is common to use nickel column with imidazole elution. Sample loading in the presence of a low concentration of imidazole and a moderate concentration of salt significantly reduced nonspecific binding of contaminating proteins (Mackin 1999). As high concentrations of urea are compatible with immobilized metal ion affinity chromatography (IMAC), it is useful to purify denatured proinsulin from inclusion bodies.

**Table 6:** Affinity chromatography in proinsulin (PI) purification

Fusion protein	Media	Comments	References
Denatured poly-histidine/ PI fusion protein	Ni^2+^–activated chelating Sepharose	Washed with buffer A: 20 mM NaPO_4_, pH 7.4, 500 mM NaCl, 20 mM imidazole, 8 M urea Elution with linear gradient (10 CVs), ending with buffer A containing 500 mM imidazole	Mackin (1999)
Histidine-tagged denatured PI	Ni–NTA His•Bind Superflow	Capture Histidine-tagged peptide Binding and elution buffers contain 8 M urea Elution with 0.15 M imidazole, pH 7.5 Yield: 77.8%; Purity: > 79% → 97.6%	Yuan et al. (2015)
Histidine-tagged sulfonated PI	Ni-chelating Sepharose FF	Stepwise gradient elution with 8 M urea and 0.08 M imidazole	Astolfi et al. (2004)
Histidine-tagged sulfonated PI	NTA column	Eluted according to a standard protocol (Qiaexpression, Qiagen)	Redwan et al. (2007)
Hexahistidine-tagged sulfonated PI	Ni-IDA-Sepharose	Binding and elution buffers contain 6 M urea Elution with 0.1 M imidazole Yield: 90% Purity: 85–95% (order of chromatography affects purity) High selectivity to poly-His sequence	Tikhonov et al. (2001)
Histidine-tagged renatured PI	IMAC	Elution with a 15 CV gradient from 0 to 400 mM imidazole Purity: 92%	Zimmerman and Stokell (2010)
Z-PI (secreted)	IgG Sepharose 6 Fast Flow	Equilibration with Tris-Saline-Tween (TST) buffer (1 mM EDTA 25 mM Tris–HCl, pH 8, 0.2 M NaCl, 0.5 mL Tween 20) Elution with 0.5 mM acetic acid, pH 2.8	Mergulhao et al. (2004)
ZZ-PI (secreted)	IgG Sepharose 6 Fast Flow	Equilibration with Tris-Saline-Tween (TST) buffer (1 mM EDTA 25 mM Tris–HCl, pH 8, 0.2 M NaCl, 0.5 mL Tween 20) Elution with 0.5 mM acetic acid, pH 2.8	Mergulhao et al. (2001)
ZZ-PI	IgG Sepharose	Binding buffer contains 0.1 M glycine–NaOH, BME Elution with 0.3 M acetic acid, pH 3.1 Yield: 90% ~ 70% of ZZ-R-proinsulin was recovered in monomeric form	Nilsson et al. (1996)

**Table 7:** A list of fusion tags used in insulin purification and their advantages

Tags	Advantages	Example references
Five leader peptides engineered with different sequences	Improved peptide expression levels Improved refolding yield, because the leader peptide affects protein conformation and hydrophobicity Simultaneous removal of the N-fused sequence and the C-peptide by trypsin in a single step	Min et al. (2011)
Polyhistidine	For purification by metal chelate affinity chromatography (Astolfi et al. 2004; Mackin 1999; Redwan et al. 2007; Tikhonov et al. 2001; Yuan et al. 2015; Zimmerman and Stokell 2010) As an easily observed indicator, using either RP-HPLC or SDS-PAGE to show that the N-terminal methionine and the rest of the poly-His affinity tag has been removed from the fusion protein (Mackin 2014) To reduce the rate of peptide degradation by stabilizing the expressed peptide and prevent N-terminal degradation (Cowley and Mackin 1997; Mackin 2014) Improved peptide expression levels due to increased stability from (His)_10_ tag of leader peptide and the tendency to stay at the exterior of the proinsulin molecule because of its polarity (Sung et al. 1986)	Astolfi et al. (2004) Cowley and Mackin (1997) Mackin (1999) Mackin (2014) Mackin and Choquette 2003) Redwan et al. (2007) Sung et al. (1986) Tikhonov et al. (2001) Winter et al. (2002a, b) Yuan et al. (2015) Zimmerman and Stokell (2010)
Two synthetic IgG-binding domains (ZZ) derived from staphylococcal protein A	For purification by IgG-affinity chromatography ZZ-tail is highly resistant to proteolysis ZZ-tail contains no cysteine residues that could cause unwanted disulfide bridges Improved peptide expression levels Simultaneous removal of the N-fused sequence and the C-peptide by trypsin in a single step, without cleavage of the target protein The solubilizing properties of ZZ enable in vitro product refolding at high protein concentrations ZZ-tag is useful for the facile detection and quantitation of staphylococcal protein A (or its engineered domain) fusion proteins secreted to the growth medium using quantitative ELISA (Mergulhao et al. 2001)	Mergulhao et al. (2001) Mergulhao et al. (2004) Nilsson et al. (1996)
One synthetic IgG-binding domains (Z) derived from staphylococcal protein A	Z-tail is highly resistant to proteolysis Using a single Z domain instead of the ZZ domain as a fusion partner led to the recovery of a 1.6-fold higher amount of PI after cleavage of the fusion tag, although no effect of the molecular size was seen on the secretion efficiency of the system	Mergulhao et al. (2004)

A well-designed fusion tag is desirable for both upstream and downstream processes. Besides the obvious function for purification, the fusion tag can improve the stability of recombinant human insulin/analogue. This is especially useful given that the proinsulin is a short-length peptide and is prone to degradation (Min et al. 2011). Other than that, an appropriately engineered fusion tag can achieve an improvement in proinsulin peptide expression levels (Min et al. 2011; Nilsson et al. 1996). The introduction of hydrophilic amino acids to fusion tag enhanced the solubility of the molecule and resulted in an improvement in renaturation yield downstream (Min et al. 2011; Nilsson et al. 1996).

Gene fusions may be tricky to handle in downstream processing, owing to the fact that the fusion tail has to be cleaved off and removed. To avoid using the toxic CNBr to cleave fusion proteins, it is possible to engineer lysine and arginine linkers that serve as trypsin cleavage site to join the fusion protein and proinsulin together. In this case, a simultaneous removal of both C-peptide and N-fused sequence can be achieved in just a single enzymatic conversion step (Min et al. 2011; Nilsson et al. 1996). If the fusion tag has a molecular weight and hydrophobic properties similar to the insulin molecule, after enzymatic conversion, the two molecules will be poorly resolved during analyses such as on RP-HPLC and SDS-PAGE. Finally, the cost of resin also constitutes an important factor for consideration. The AC resin is generally more expensive than IEX resin. It may be cost-effective to switch to IEX as the capture step if the latter has equal or better purification performance. Nonetheless, the fusion tag can still be preserved for the aforementioned benefits other than for affinity binding.

#### Ion-exchange chromatography (IEX)

IEX involves the separation of ionizable molecules based on their total charge. As the human insulin/analogues and its precursor have both anionic and cationic groups, it is possible to use anion exchange chromatography (AEX) and/or cation exchange chromatography (CEX) for purification. The significant negative charge of *S*-sulfonated fusion protein can bind strongly to AEX. However, negatively charged non-protein impurities such as nucleic acids can also compete for binding. Therefore, in some instances, it is advantageous to use CEX. Table 8 provides a list of applications which have reported using IEX in the purification of human insulin/analogue and its precursor. IEX is a versatile technique that can purify either a folded insulin/insulin precursor or a sulphonated proinsulin in denaturing condition. Commonly, IEX is adopted as the capture step of precursor insulin or as the first step after enzymatic cleavage. Its high binding capacity and the possibility of including multiple wash steps enable the removal of a large number of contaminants which may complicate subsequent purification. These contaminants comprised of host cell impurities (protein, DNA, endotoxin), proteolytic enzymes, and product impurities produced during enzymatic digestion (excised fragments and miscleavages).

**Table 8:** Ion-exchange chromatography (IEX) in proinsulin (PI) and insulin purification

Protein	AEX/CEX	Media	Comments	References
Folded preproinsulin	AEX	DEAE-Sepharose fast flow/ Source 30 Q	Flow-through mode at pH 8.3, 6.1 mS/cm, in which the preproinsulin was not bound to the gel but washed through the column with the permeate The higher molecular weight impurities were adsorbed	Thurow et al. (2010)
Folded preproinsulin	CEX	Source 30 S	Elution with linearly increasing NaCl gradient	Thurow et al. (2010)
Refolded PI	AEX	Q-Sepharose Fast Flow	Equilibration with 20 mM glycine–NaOH, pH 10.0 NaCl elution in 20 mM glycine–NaOH, pH 10.0	Chen et al. (2016)
Sulfonated PI	AEX	DEAP-Spheronit	NaCl elution in 7.5 M urea Yield: 95% Purity: 70–95% (order of chromatography affects purity)	Tikhonov et al. (2001)
Sulfonated PI	AEX	Mono-Q HR	Binding and elution buffers contain 7 M urea NaCl elution	Cowley and Mackin (1997)
Sulfonated PI	AEX	Q-Sepharose Fast Flow	NaCl elution in 8 M urea	Castellanos-Serra et al. (1996)
Sulfonated PI	CEX	*S*-Sepharose	Eluted at a rate of 3 mL/min using a linear gradient of 0.5 M NaCl in 7 M urea/20 mM formic acid buffer (pH 4.0) for 50 min	Redwan et al. (2007)
Sulfonated PI	CEX	SP Sepharose Fast Flow	NaCl elution Yield: 90%	Petrides et al. (1995)
Insulin	AEX	DEAE	Purification after citraconylation and trypsin digestion step	Mikiewicz et al. (2017)
Insulin	AEX	DEAE-Sepharose	Purification after citraconylation and trypsin digestion step Elution with Tris pH 8.6 and 30% isopropanol (conductivity 6 mS)	Zieliński et al. (2019)
Insulin	AEX	Q	Purification after citraconylation and trypsin digestion step	Mikiewicz et al. (2017)
Insulin	AEX	Q-Sepharose	Elution with Tris pH 8.6 and 30% isopropanol (conductivity 3 mS)	Zieliński et al. (2019)
Insulin	AEX	Source 30Q	The load is in 30% ethanol, pH 7.5, < 3 mS/cm and contains Zn-ions in an amount of 2 zinc atoms per six insulin molecules Elution with ammonium acetate, ethanol, triethanolamine at pH 6.4, 6.8 and 7.2 At pH 6.4, the peak is fronting (flat front and steep tail), and a baseline separation is seen between the impurity and the product peak Yield: > 90%	Mollerup and Frederiksen (2016)
Insulin	CEX	BioSepra CM	Loading diluent and elution solution contain hexylene glycol 2 washes with NaCl Isocratic elution with NaCl Yield: 60–85%; Purity: > 90%	Coleman et al. (2019)
Insulin	CEX	Capto SP ImpRes	Subsequent purification after enzymatic conversion Binding buffer: Na acetate buffer pH 4, 10% ethanol Elution with 47.5% ethanol and 128 mM NaCl Yield: 102%; Purity: 63–94%	GE application note 29–0018-56 AB
Insulin	CEX	SP Sepharose Fast Flow	Capture of glargine insulin NaCl elution	Hwang et al. (2016)
Insulin	NA	NA	NaCl elution Yield: 95%	Petrides et al. (1995)

*NA* information not available

IEX is characterized by a charged surface on stationary phase, and the use of buffer, salt, and pH control on mobile phase composition. After the impure protein sample was loaded onto an IEX column, the column is washed to remove undesired proteins and other impurities. Using either a salt or pH gradient, the protein of interest is then eluted. It is more common to elute the insulin precursor/insulin with sodium chloride compared to using a pH gradient (Table 8). In salt gradient elution, the salt ions compete with bound proteins for the charged functional groups on the resin. Proteins with many charged groups will elute at high salt concentrations and thus have greater retention times. On the other hand, proteins with few charged groups will elute at earlier retention times. In pH gradient elution, the number and type of ionizable amino acid side chain groups will determine the charge on the protein. At the protein’s isoelectric point (pI), the net charge of the protein becomes zero. Protein elution occurs at the point when the pH gradient meets their pI as the protein no longer have a net charge that allows binding to the resin. An increasing pH gradient is used to elute protein from a cation exchange resin, whereas a decreasing pH gradient is used to elute proteins from an anion exchanger.

It is important to take into consideration the pI of the insulin or insulin analogue during IEX purification as it will influence the choice of resin. Insulin analogue may have a pI that is different from the native insulin. For example, the pI of native insulin is 5.4–5.6; however, the pI of an acid-stable insulin analogue (e.g., insulin glargine) is about 6.7–7.0 (Coleman et al. 2019). A weak cation exchanger may be applicable for insulin analogues with a pI greater than the pI of native insulin, whereas a strong cation exchanger is used for insulin analogues with a pI similar to that of native insulin (Coleman et al. 2019). A strong cation exchanger is ionized across a wide range of pH levels, whereas a weak cation exchanger is ionized within a narrower pH range and they start to lose their ionization below pH 6.

The stability and solubility of insulin precursor/insulin is another factor for consideration. Insulin/insulin analogue is soluble in acidic condition, but it has a limited solubility close to its pI and neutral pH (Sigma Aldrich 2021; Wintersteiner and Abramson 1933). In addition, the insulin molecule is unstable at both the extremes of pH. At a high pH, there is an increased risk of deamidation and aggregation (Helmerhorst and Stokes 1987). On the other hand, a pH that is overly acidic also poses the risk for deamidation (Brange and Langkjœr 1993). Hence, a suitable pH range for purification of insulin with CEX is typically set at around pH 3 to 4. In a salt gradient elution, the pH can be used to refine the eluted peak resolution. An optimal pH should provide maximal binding of insulin peptide and minimal binding of contaminants.

A vital consideration for IEX purification of insulin precursor/insulin lies in the buffer composition during loading, washing, and elution. Acetic acid is a preferred buffer in CEX as it has been shown to reduce the risk of insulin fibrillation (Whittingham et al. 2002). The addition of organic solvents/modifiers to the eluent can help to maintain good protein solubility. For example, the addition of ethanol may increase the solubility of insulin as the molecule is relatively hydrophobic (GE application note 29-0018-56 AB 2012; Mollerup and Frederiksen 2016). The presence of ethanol may improve chromatographic performance, such as by decreasing elution volume and increasing yield (Heldin et al. 2014). The wash buffers should be optimized with respect to pH and salt content to ensure maximal removal of impurities.

Some impurities may be very challenging to remove, especially if they carry the same charge as the target peptide. It has been found that in the presence of di- or polyvalent metal ions in binding buffer, the insulin peptides are capable of self-association or having structural change (Mollerup and Frederiksen 2016). This results in an improvement in the control of peak shapes and leads to a good resolution of the protein of interest from closely related impurities. For example, the addition of Zn^2+^ to the binding buffer could result in a fronting peak shape of the insulin peptide, which have moved away from the earlier eluting insulin-related impurities (Mollerup and Frederiksen 2016). Also, a steep insulin peak tail makes it possible to obtain a very concentrated pool of purified insulin. This is advantageous in industrial production scale, in which the load can be increased while maintaining the same capacity to remove impurities.

#### Reversed-phase chromatography (RP)

RP involves separation of molecules based on the hydrophobic interactions between ligands attached to the stationary phase and solute molecules in the mobile phase. There is a difference in mechanism by which polypeptides interact with the RP surface, as compared to the small molecules (Carr 2016). In the separation of small molecules, there is continuous partitioning of the molecules between the mobile phase and the hydrophobic stationary phase. On the other hand, polypeptides are too big to partition into the stationary phase. The hydrophobic “foot” of the polypeptide remains adsorbed to the hydrophobic surface of the stationary phase up to the point a specific organic modifier concentration is reached, and this will desorb the polypeptide.

Table 9 presents reported applications of RP in the human insulin/analogue purification scheme. RP is usually placed after the enzymatic conversion of proinsulin to insulin. Following enzymatic digestion, the purification of insulin or insulin analogues usually requires two to three orthogonal chromatographic purification steps (Table 5). Purification with RP complements IEX and SEC by providing selectivity based on differences in hydrophobicity.

**Table 9:** Reversed-phase chromatography (RP) in proinsulin (PI) and insulin purification

Protein	C4/C8/C18	Media	Comments	References
Renatured PI	C4	Vydac C4	Equilibration: 4% ACN, 0.1% TFA Elution with a linear gradient of increasing ACN (0.88%/min) Purity: 90%	Cowley and Mackin (1997)
Renatured PI	C4	Vydac C4	Buffer A = 0.1% TFA in water, Buffer B = 0.1% TFA in 20% water/80% acetonitrile Linear gradient increasing to 60% B over 40 min	Mackin (1999)
Renatured PI	Polystyrene-divinylbenzene matrix	SOURCE 15RPC	Buffer A = 0.1% TFA in water, B = 0.1% TFA in 80% acetonitrile/20% water Gradient elution of 30–50% B over 50 min 60% recovery Recovered 95–98% pure DKP-hPI	Mackin and Choquette (2003)
Insulin	C8	C8 prep HT	Purification of glargine insulin Elution with a linear gradient of 15% to 36% ACN (0.88%/min) Final product purity: 98.11% (1.96% desamido insulin)	Hwang et al. (2016)
Insulin	C8	Kromasil C8	Dissolving buffer contains acetone or ACN Elution with n-propanol in buffered solvent comprising zwitterions, e.g., glycine or betaine Product is virtually free from proteases and insulin acetylated at position A9	Dickhardt and Unger (1997)
Insulin	C8	Kromasil C8	Elution with a gradient of 0 ± 22% buffer B (50% isopropanol in water, 1.5 mS ammonium sulfate, pH 3.0)	Mikiewicz et al. (2017)
Insulin	C8	Kromasil C8	Equilibration: 30% ACN, 0.25% pentafluoropropionic acid (PFPA) Elution with a gradient of 30% to 50% ACN, 0.25% PFPA Yield: 54%	Nilsson et al. (1996)
Insulin	C8	Kromasil C8	Elution with a linear gradient of isopropanol	Watson et al. (2018)
Insulin	C8	Zorbax Process grade C8	Load are partially purified human insulin zinc crystals Elution in linear gradient of 0.25 M acetic acid (eluent A) to 60% ACN (eluent B) Yield: 82%; Purity: 98.5% Ideal pH is in the region 3.0 to 4.0, which is below the isoelectric point of 5.4 Acidic mobile phase provided resolution of insulin from structurally similar insulin-like components while promoting insulin solubility	Kroeff et al. (1989)
Insulin	C18	ACE 5 C18-300	Equilibration: 0.2 M sodium sulfate pH 2.3 and ACN in ratio of 4.5:1 Elution with 0.2 M sodium sulfate pH 2.3 and ACN in ratio of 1:1	Zieliński et al. (2019)
Insulin	C4/C8/C18	C4/C8/C18	Isocratic or a shallow gradient elution with ACN in the presence of 200 mM sodium sulfate and 0.16% phosphate	Zimmerman and Stokell (2010)
Insulin	C8/C18	Kromasil C8; Lichrospher Select B, C8; Zorbax Pro10, C8; Nucleosil C18; Nucleosil C18-P	Elution with n-propanol/ethanol gradient in buffered solvent, in the presence of zwitterions, e.g., glycine, glutamic acid or glycine betaine The solvent mixture is within about one pH unit above or below the isoelectronic point of the insulin or insulin derivative to be purified	Dickhardt et al. (1993)
Insulin	NA	Kromasil	Mobile phase: ACN/ 0.2 M ammonium acetate buffer, pH 4 Gradient: 0 min: 22%, 60 min: 32% ACN Co-elution of human insulin with impurity	Kromasil (2021)
Insulin	NA	NA	Elution with 25% ACN, 1.5% acetic acid, 73.5% water Yield: 95%	Petrides et al. (1995)

*NA* information not available, *ACN* acetonitrile, *TFA* trifluoroacetic acid

RP is typically placed late in the overall purification scheme (e.g., after IEX) to allow the preceding step to remove a majority of the host cell-related impurities. This will be beneficial in prolonging the effective lifetime of RP stationary phase as it is prone to fouling as a result of irreversible binding or low solubility of contaminants (Kroeff et al. 1989). This is critical as the silica-based packings have pH limitations which prevent the use of pH extremes for chromatography clean-up and regeneration.

The high selectivity and resolving capability of RP is the foundation for its widespread use as a “polishing” step in the purification of human insulin/analogue. A key challenge with the purification of human insulin/analogues is the downstream elimination of process-related impurities arising from insufficient cleavage of the insulin precursor molecule or miscleavage. Such product impurities include C-peptide, N-terminal signal sequences, dipeptides, aggregated insulin, misfolds, miscleaves, deamidated insulin, and any residual proinsulin (Watson et al. 2018). Often, minor modifications on the insulin molecule result in the generation of A21 desamido insulin (hydrolysis of A21 asparagine to aspartic acid), B30 des-threonine insulin (deletion of B30 threonine), or derivatization of amines by formylation or carbamoylation (Petrides et al. 1995). The most significant ones among these contaminants are A21 desamido insulin and B30 des-threonine insulin (Balcerek et al. 2018). As des-threonine insulin carries the same net charge as insulin, IEX is not ideal in resolving the two molecules. Nonetheless, the molecules’ small differences in hydrophobicity make RP useful in removing the contaminant. The resemblance in chemical structure between human insulin/analogue and its product-related impurities, which may differ by only a single amino acid, has posed an uphill task in the purification of human insulin/analogue. However, there have been many reports which have demonstrated the capability of RP to resolve such close-related insulin variants and fragments generated after trypsin digestion (Table 9).

RP ligand with linear aliphatic hydrocarbon chain of eighteen (C18), eight (C8), or four (C4) carbons can be used in the purification of human insulin/analogue, with the most common one involving C8, followed by C18 and C4 (Table 9). C18 is usually preferred for small hydrophilic peptides in the range of 2–3000 Da, whereas C4 is most suitable for the separation of proteins, and large or hydrophobic peptides (Carr 2016). The size of insulin (at about 6000 Da) after digestion and its relative hydrophobicity makes C8 column, which is an intermediate between C4 and C18, ideal for insulin separation. It is best to try several different hydrophobic phases to ascertain which has the best selectivity for that specific mixture of peptides generated from protein digestion. Variation in the types of insulin analogues and fragments generated post trypsinization, may result in some differences in their “hydrophobic foot”. Also, subtle changes in RP adsorbent surfaces may result in differences in RP selectivity (Carr 2002).

Due to the highly hydrophobic nature of RP adsorbents, an organic solvent as the mobile phase has to be used in peptide elution. The ability of human insulin/analogue to tolerate such a harsh elution condition makes RP a suitable tool for purification. The organic solvent most commonly used in human insulin/analogue RP purification is acetonitrile (Table 9). Its long history of use, high volatility and thus easy removal after purification, low viscosity and thus low back pressure, transparency to low wavelength UV light, and ability to provide good chromatographic selectivity and peptide solubility make it preferable over many other solvents such as ethanol, isopropanol, and acetone (Carr 2016; Kroeff et al. 1989). There are also reports on the usage of isopropanol for insulin purification (Dickhardt et al. 1993; Dickhardt and Unger 1997; Mikiewicz et al. 2017; Watson et al. 2018). Isopropanol, which is less polar than acetonitrile, may enhance recovery or elution of hydrophobic insulin molecule.

The RP mobile phases used in insulin purification are generally adjusted to a low pH. At a low pH, protonation of the carboxylic acid groups on the side chains of glutamic and aspartic acids, and on the carboxy terminal group will occur and this will make the protein more hydrophobic (Carr 2016). This will increase the retention of peptides. Also, an acidic mobile phase typically elutes all the insulin derivative impurities after the insulin peak and this will simplify the collection step (Petrides 1995). In contrast, a buffer with mildly alkaline pH causes the elution of derivatives to be on either side of the parent insulin peak, thus sacrificing on the yield recovery and purity of the insulin pool.

An ideal pH for human insulin/analogue purification on RP is in the range of 3.0–4.0, as this pH range is under insulin’s isoelectric point to provide for good solubility (Kroeff et al. 1989; Ladisch and Kohlmann 1992). In addition, the mildly acidic conditions will reduce the formation of monodesamido (B-3) insulin generated at pH 7 or slightly above (Brange 2012; Kossiakoff 1988). As the typical pH range for RP on a silica-based packing is pH 2–8 (Verma 2021), the mildly acidic mobile phase will be within the pH tolerance of stationary phase. Stationary phase stability is critical in avoiding contamination of the human insulin/analogue with these potential breakdown products and ensuring a good column lifetime (Kroeff et al. 1989). Despite the advantages of using mildly acidic conditions, it is critical to perform the RP purification as rapidly as possible and avoid prolonged storage of human insulin/analogue in acidic condition to minimize deamidation (Ladisch and Kohlmann 1992).

It is important to select an appropriate acidic buffer as mobile phase in insulin purification. Insulin can form fibrillation (elongated insoluble aggregates) at a high salt concentration (Chatani et al. 2014; Muzaffar and Ahmad 2011). Thus, the mobile phase should be capable of maintaining a stable pH without the addition of salts. Acetic acid-containing mobile phase is a good option, because acetic acid is a weak acid that buffers in the region of pH 3 without the need for other salts (Kroeff et al. 1989; Petrides et al. 1995). Also, insulin can be readily crystallized from the acetic acid buffer by adding zinc chloride. A proprietary ion-pairing chromatography method on RP was reportedly able to improve the recovery of pure insulin collected as the des-threonine insulin impurity is displaced away from the insulin peak (Kromasil 2021).

It is also common to elute human insulin/analogue and impurities by a gentle linear change in gradient compared to using an isocratic elution (Table 9). This is due to a few reasons. First, the sensitivity of polypeptide retention to subtle changes in the organic modifier concentration makes isocratic elution challenging, due to the fact that any small changes in modifier concentration can significantly affect protein retention (Carr 2016). To separate impurities from the insulin peak in isocratic elution, the concentration of the organic modifier must be maintained very precisely in every batch. Second, a gradient elution of polypeptide yields much sharper peaks than in an isocratic elution (Carr 2016). A concentrated pool obtained from gradient elution makes handling and processing of human insulin/analogue much easier. Finally, a gradient elution can minimize the run time as compared to using isocratic elution in RP (Healthcare GE 2006).

#### Hydrophobic interaction chromatography (HIC)

HIC, similar to RP, separates molecules based on their hydrophobicity. However, unlike RP, HIC adsorbents are not as hydrophobic. Thus, organic solvents are not a prerequisite for successful elution. HIC is useful for purifying sensitive proteins as their biological activities are preserved under less denaturing chromatographic conditions.

There are many methods for HIC elution optimization—linear or stepwise decreasing gradient of a salt and/or zwitterion, a pH gradient, a temperature gradient, or a combination of the before-mentioned (Mollerup and Frederiksen 2016). It is also possible to adopt an elution gradient of a calcium chelating compound (e.g., citrate, EDTA, and malonate) or a solvent less polar than water (e.g., aqueous solutions comprising ethanol, 2-propanol, and polyethylene glycol (PEG)) (Mollerup and Frederiksen 2016). Using the salting-out effect, the retention of peptide can be modulated by adjusting the salt concentration (Johansson et al. 2015). It is however important to select a suitable salt for proinsulin purification on HIC, as the type of salt chosen for binding and elution can have an effect on product yield and purity (Bai et al. 2003).

Some of the example applications of HIC in proinsulin purification are presented in Table 10. As a gentler purification technique as compared to RP, it may be suitable to use HIC following proinsulin renaturation, so that the peptide conformation is maintained (Petrides et al. 1995). The use of HIC for a simultaneous purification and refolding of proinsulin has also been reported (Bai et al. 2003).

**Table 10:** Hydrophobic interaction chromatography (HIC) in proinsulin (PI) purification

Protein	Media	Comments	References
Denatured PI	Own produced columns with end groups containing PEG 600 and phenyl	Simultaneous purification and refolding of proinsulin Buffer A: 3.0 M ammonium sulfate, 0.05 M potassium dihydrogen phosphate, pH 7.0 Buffer B: 0.05 M potassium dihydrogen phosphate, pH 7.0 Gradient elution mode Flow rate: 1 mL/min	Bai et al. (2003)
Renatured PI	NA	Purification after renaturation step Yield: 90%	Petrides et al. (1995)

NA: Information not available

Compared to RP, there are only few reports available on the use of HIC in human insulin/analogue production. Human insulin/analogue polishing step requires an extremely high-resolution separation of closely related impurities that have only minor differences in hydrophobicity. This may make RP preferable over HIC as RP is known to be the highest resolution chromatography technique available (Healthcare GE 2006). To ensure insulin adsorption on a HIC column, the feed should have the same high salt concentration as the mobile phase during elution. Therefore, another disadvantage of HIC is that diluted large insulin feed volume had to be used as the insulin solubility was reduced due to the high salt content in the feed. It has been reported that column load for HIC with butyl adsorbent was 0.01–2.4 g insulin/L column and 0.5–1.3 g insulin/L column for the phenyl adsorbent, which paled in comparison with the RP columns (2.9–11.7 g insulin/L column) (Johansson et al. 2015).

#### Size exclusion chromatography (SEC)

SEC, also known as gel filtration, is the mildest of all the chromatography techniques. SEC separates molecules by differences in size as they pass through the resin. There is no binding of the molecules to the chromatography resin, unlike chromatography techniques such as AC and IEX. SEC is typically used in the polishing step in human insulin/analogue purification (Table 5). It has also been adopted in protein-folding liquid chromatography (PFLC) for simultaneous purification and renaturation of proinsulin (Yuan et al. 2015). During elution with refolding buffer, the folded proinsulin was separated from the misfolded and aggregated forms. Table 11 presents examples of reported application of SEC in proinsulin and human insulin/analogue purification.

**Table 11:** Size exclusion chromatography (SEC) in proinsulin and insulin purification

Protein	Media	Comments	References
Denatured proinsulin	TSKgel G2000SWXL	Buffer A: 8 M urea, 0.5 M NaCl, 20 mM PBS, 10 mM imidazole Linear gradient (0–100%) elution with mobile phase B 2.0 M urea Purity: > 79% → 93.7% Refolding and simultaneous purification	Yuan et al. (2015)
Insulin	NA	Elution with acetic acid solution Yield: 90%	Petrides et al. (1995)

*NA* information not available

#### Mixed-mode chromatography (MMC)

In recent years, MMC is becoming increasingly popular in human insulin/analogue purification. MMC utilizes more than one form of interaction between the stationary phase and analytes to achieve their separation (Zhang and Liu 2016). The multiple modes of interaction include affinity, ion exchange, size exclusion, hydroxyapatite, and hydrophobic interactions. The mixed-mode ion exchangers give a different selectivity as compared to the conventional ion exchangers. It also enables the possibility of proteins binding at a high salt condition, which eliminates the need for desalting or dilution prior to carrying out MMC. Table 12 presents examples of reported application of MMC in proinsulin and human insulin/analogue purification.

**Table 12:** Mixed-mode chromatography (MMC) in proinsulin (PI) and insulin purification

Protein	Resin ligand functional group (interactions)	Media	Comments	References
Sulfonated PI	Multimodal weak cation exchanger (ionic interaction, hydrogen bonding, and hydrophobic interaction)	Capto MMC	Binding and elution buffers contain 8 M urea pH step elution from 5.2 to 8 Yield: 96%; purity: 82% Ability to bind sample without prior dilution	GE app. Notes 28-9966-22 AA (2012)
Insulin	Multimodal strong anion exchanger (ionic interaction, hydrogen bonding and hydrophobic interaction)	CaptoAdhere	Elute with a buffer of pH 3.4–3.6 Two wash steps at a pH higher than the elution pH to remove the majority of the impurities from enzymatic digest	Watson et al. (2018)

#### pH precipitation

The precipitation method has been adopted in human insulin/analogue downstream processing for a crude and rapid separation of human insulin/analogue from other impurities. This method can also be used to concentrate the protein for further purification steps.

Purification and concentration of the solubilized protein from inclusion bodies can be achieved by pH precipitation. As a protein has the lowest solubility at its isoelectric point (pI), it will precipitate out of the solution at its pI. Following that, centrifugation can be performed to recover the protein of interest. For example, solubilized fusion protein can be precipitated by lowering the pH to 5.0, centrifuged, and then re-dissolved in an alkaline buffer at pH 8.5 to enhance the purity of the solubilized protein (Astolfi et al. 2004). After sulfitolysis reaction, the *S*-sulfonated preproinsulin can be precipitated by adjusting the pH to 4.5, centrifuged, and the washed pellets can be solubilized again by raising the pH to 10.6 (Kim et al. 2015; Min et al. 2011). The pH precipitation step can also be carried out after proinsulin refolding (Hwang et al. 2016; Min et al. 2011; Thurow et al. 2010). The pH of the refolded proinsulin solution can be lowered to pH 4.5–5.5 to precipitate out the impurities, misfolded proinsulin, lipids, and other proteins that hinder subsequent enzymatic conversion and chromatography purification. The supernatant containing correctly folded proinsulin is collected by centrifugation. It is important to determine the exact pH required to precipitate the impurities, while maintaining the correctly refolded proinsulin in solution.

#### Zinc crystallization

In the presence of zinc ions, insulin is capable of self-association to form hexamers, di-hexamers, or bigger complexes of insulin peptide (Mollerup and Frederiksen 2016). The hexamer consists of three dimers closely associated through interacting with two zinc ions at its core positioned on the threefold axis. The aptitude of insulin to form crystals readily can be utilized in the purification scheme to isolate insulin from impurities that do not co-crystallize with it (Mollerup et al. 2010). Zinc crystallization is a convenient and efficient method to concentrate and hold the insulin temporarily prior to further processing (Kroeff et al. 1989). The hexameric form also protects insulin from physical and chemical degradation during storage (Mollerup and Frederiksen 2016). In proinsulin purification, the addition of 0.1 M to 0.5 M zinc chloride solution in between chromatography purification steps has been reported to precipitate out the eluted proteins (Redwan et al. 2007). After decitraconylation, 1 M zinc chloride solution was added to the insulin-containing sample and the pH was adjusted to 5.9 to precipitate out insulin (Zieliński et al. 2019). Zinc crystallization can be performed as soon as possible after the RP purification step to remove insulin from the acidic environment to minimize deamidation (Brange 1994).

### Refolding

The insulin hormone is comprised of two polypeptide chains, an A chain of 21 amino acids and a B chain of 30 amino acids. These two chains are connected by two interchain disulfide bridges (A20-B19 and A7-B7). The A chain also features one intra-chain disulfide bridge (A6-A11). In vitro*,* the proinsulin is believed to spontaneously fold into its native structure through preferred kinetic intermediates (Jia et al. 2003). The refolding pathway of human proinsulin has been studied by capture and analysis of their folding intermediates. Although several intermediates with three disulfide bonds have been identified and may interconvert via disulfide rearrangements (Qiao et al. 2003; Hua et al. 2002), the formation of the A20-B19 disulfide bridge appears to be the key step in refolding process and is critical to maintain the native structure of the proinsulin (Guo et al. 2008; Qiao et al. 2006). Unsurprisingly, a study on three disulfide deletion mutants of human insulin revealed that the deletion of the A20-B19 disulfide bond had the greatest influence on the protein structure, while the deletion of A6-A11 disulfide bond had the least effect (Chang et al. 2003).

During in vitro renaturation, the high concentration of denaturant has to be removed to facilitate disulfide-bond formation and correct folding of proinsulin to its native form (Harrison et al. 2015). Dilution of the solubilized protein in refolding buffer is by far the most popular choice in insulin production. Other methods to refold proinsulin include protein-folding liquid chromatography (PFLC) (Yuan et al. 2015) and dialysis (Mackin 1999). The optimized conditions reported for proinsulin refolding via dilution are summarized in Table 13. The success of refolding depends in part on the composition of refolding buffer. Glycine–NaOH is the most common buffer used in proinsulin refolding as it buffers in the range of pH 8.6–10.6. The high pH leads to faster disulfide-bond formation and shuffling (Min et al. 2011). Refolding performed at pH 10.5 using 0.5 mM cysteine and 4.5 mM cystine as redox couple yielded a higher refolding yield (60%) compared to refolding at a lower pH of 8 which yielded only a 20% yield (Winter et al. 2002b).

**Table 13:** Reported optimized conditions for proinsulin (PI) refolding via dilution

Protein	Refolding yield (%)	Refolding duration	Refolding buffer	pH	Urea/ GdnHCl (M)	EDTA (mM)	Oxidants/reductants/redox couples	Other additives	Temp. (°C)	Protein concentration (mg/mL)	References
rhPI	99	18–19 h	12 mM NaHCO3	10.8	–	0.2	–	–	7–8	NA	Zieliński et al. (2019)
rhPI	85	12 h	NA	NA	–	–	BME (1.5 mol/mol of SO_3_^2−^)	–	8	< 1 mg/mL	Petrides et al. (1995)
rhPI	43	1 h	10 mM Tris/10 mM glycine	7.5	43 mM GdnHCl	1	1.8 molar ratio of GSH/GSSG	10 µM Vectrase-P	25	10 µM	Winter et al. (2002a)
rhPI	NA	16 h	50 mM glycine/NaOH	10.5	–	–	BME (1.5 mol per mol of cysteine * S*-sulfonate)	–	4	2 mg/mL	Cowley and Mackin (1997)
rhPI	NA	Overnight	50 mM glycine/NaOH	10.5	–	1	1 mM GSH, 1 mM GSSG	–	4	0.1 mg/mL	Mackin and Choquette (2003)
rhPI	NA	16 h	10 mM glycine	10.6	0.6 M urea	–	0.3 mM BME	–	15	1.5 mg/mL	Son et al. (2009)
HGH-PI fusion protein	NA	24 h	1% glycine, 50 mM Tris–HCl, 100 mM NaCl	9.3	3.2 M urea	–	1 mM GSH, 1 mM GSSG	5% glycerol, 0.2% PEG	4	NA mg/mL	Leng et al. (2013)
His_8_–Arg–proinsulin	60–70	10–30 min	10 mM Tris, 10 mM glycine	10–11	3 M GdnHCl or 4 M urea	1	0.5 mM cysteine, 4.5 mM cystine	–	15	0.5 mg/mL	Winter et al. (2002b)
Hybrid insulin analogue precursor	NA	18 h	2 mM NaHCO3	11.2	–	0.2	–	–	NA	NA	Mikiewicz et al. (2017)
Interleukin-2-proinsulin * S*-hexasulfonate	80	6 h	50 mM glycine	10.5	–	1	BME (1.5 eq. per eq. of * S*-sulfonate)	–	4	0.1 mg/mL	Castellanos-Serra et al. (1996)
Novel PI	NA	≥ 48 h	5 mM CAPS	10.5	–	–	Equal amount of GSSG to the initial amount of reducing agent	–	2–10	0.5 mg/mL	Zimmerman and Stokell 2010)
PI aspart	80	Overnight	20 mM glycine–NaOH	10	2 M urea	–	10 µM oxidant of CASeSeCA	–	4	0.3 mg/mL	Chen et al. (2016)
PI aspart	80	Overnight	20 mM glycine–NaOH	10	–	–	5 µM of an oxidative agent of selenocystamine	0.5 M arginine	4	0.5 mg/mL	Chen et al. (2016)
Prepeptide fusion glargine	82.1	48 h	50 mM glycine	10.6	0.6 M urea	–	0.1 mM BME	–	4	0.5 mg/mL	Hwang et al. (2016)
Pure sulfonated PI	NA	18–24 h	10 mM glycine	10.0	–	–	0.5 mM cystine, 0.5 mM BME	–	4–8	0.5 mg/mL	Astolfi et al. (2004)
*S*-sulfonated fusion preproinsulin	85	17 h	50 mM glycine	10.6	0.3 M urea	–	BME (0.75 equiv. of –SH per –SSO_3_^−^)	–	12	0.5 mg/mL	Min et al. (2011)
*S*-sulfonated PI	NA	16 h	50 mM glycine/NaOH	10.5	1 M urea		1 mM BME, 0.15 mM cystine	–	4	2 mg/mL	Redwan et al. (2007)
*S*-sulfonated preproinsulin	74.2	16 h	50 mM glycine	10.6	0.6 M urea	–	0.3 mM BME	–	15	1.5 mg/mL	Kim et al. (2015)
ZZ-R-PI	NA	20 h	0.1 M glycine–NaOH	10.5	–	–	BME (18 mol/mol fusion protein)	–	4	0.8 mg/mL	Nilsson et al. (1996)

*NA* information not available, *rhPI* recombinant human proinsulin, *BME* β-mercaptoethanol, *GSH* reduced glutathione, *GSSG* oxidized glutathione, *GdnHCl* guanidine hydrochloride, *PEG* polyethylene glycol

To improve proinsulin refolding yield, chaotropic agents (e.g., urea and guanidine hydrochloride) and amino acids may be added to the refolding buffer. Arginine, low concentration of urea, and guanidine hydrochloride were reported to increase the solubility and suppress precipitation during proinsulin refolding (Qiao et al. 2003; Winter et al. 2002b). Although arginine and urea were both used to assist in proinsulin refolding, their efficacy and possible mechanism was found to be different (Chen et al. 2016). The oligomers formed with urea were of larger size than with arginine. It is important to optimize the concentrations of these reagents during the protein refolding process. The presence of 1.5 M guanidine hydrochloride in the refolding sample may reduce the aggregation of folding intermediates and this may be beneficial when folding was performed at a higher protein concentration of 1–2 mg/mL (Winter et al. 2002b). With the urea concentrations increasing from 2 to 4 M, the refolding yield of proinsulin aspart decreased from 40 to 30% due to the increase of disulfide-isomerized monomers (Chen et al. 2016). On the contrary, the refolding yield gradually increased to 50% with increasing arginine concentrations up to 1 M. Castellanos-Serra et al. (1996) have found that urea and guanidine hydrochloride at low molar concentrations (0.5 M–2 M) had no substantial effect in folding yield of interleukin-2-proinsulin. Hydrogen peroxide, an uncommon component in refolding buffer, is shown to minimize the formation of insulin derivatives and resulted in a simultaneous improvement of the human insulin production yield (Son et al. 2008).

It was found that protein hydrophobicity could have an influence on their sensitivity against redox reagents (Min et al. 2011). The refolding process depends largely on the redox potential. It was found that all three ratios of reduced and oxidized glutathione at 2:1, 1:1, and 1:2 resulted in complete refolding of DKP-human proinsulin (Mackin and Choquette 2003). Winter et al. (2002b) found that the yield of refolding increased as the refolding buffer became more oxidizing, possibly attributed by less formation of intermediates susceptible for aggregation. Refolding with cysteine/cystine yielded about two times more native proinsulin and the process was more efficient compared to folding in the presence of glutathione. Optimum renaturation with cysteine/cystine took place under more oxidizing conditions than with GSH/GSSG. The type and concentration of oxidant, reductant, or redox couple should be optimized to get a high refolding yield. It was demonstrated that only oxidant, instead of redox pairs, was necessary to facilitate the native disulfide-bond formation for the reduced denatured proinsulin (Chen et al. 2016). An oxidative agent of selenocystamine could improve the proinsulin refolding yield up to 80% in the presence of 0.5 M arginine. BME is a common reducing agent added for appropriate disulfide-bond formation (Kyte and Doolittle 1982; Petrides et al. 1995), and its concentration has to be optimized to assure a high refolding yield (Min et al. 2011). At a higher cysteine-to-proinsulin-SH ratio, some intermediates and folded lyspro-proinsulin were reduced to proteins with thiolate anions, which caused unfolding and high yield loss (Chen et al. 2010).

It is important to reduce the intermolecular interactions during refolding to minimize protein aggregation, and this will lead to an increase in refolding yield (Chen et al. 2010; Singh et al. 2015). Several studies have shown the effects of protein concentration on refolding yields (Chen et al. 2016; Kim et al. 2015; Mackin and Choquette 2003). It is recommended to dilute proinsulin to under 1 mg/mL to prevent cross-folding of molecules (Petrides et al. 1995). It was found that the interleukin-2-proinsulin refolding yield at 800 µg/mL was 15% of the yield at 200 µg/mL, in which the latter has a refolding yield of about 80% of correctly folded proinsulin (Castellanos-Serra et al. 1996). Refolding of DKP-human proinsulin proceeded similarly well at 50, 100, and 200 μg/mL, though a concentration at 400 μg/mL failed to result in complete refolding (Mackin and Choquette 2003). A maximum refolding yield at 50% was obtained when the proinsulin protein concentration was kept at 0.5 mg/mL, compared to 30–40% yield at a protein concentration of 1–2 mg/mL (Winter et al. 2002b).

There is a wide range of temperatures (2–25 °C) reported in proinsulin refolding (Table 13). Although a change in temperature could influence the kinetic of the formation of native proinsulin, the proinsulin refolding process was not temperature-dependent and temperature had no significant effect on the final yield (Winter et al. 2002b; Zieliński et al. 2019). This could be attributed to the good thermal stability that insulin has against thermal unfolding (Winter et al. 2002b).

The refolding duration varies from as short as 10 min to more than 48 h (Table 13). The proinsulin refolding process can be monitored to completion by analyzing aliquots of the reaction mixture on RP-HPLC. Once the steady yield was reached, it would not increase or drop further as the reaction time proceeds (Castellanos-Serra et al. 1996).

Min et al. (2011) has demonstrated that the structure of leader peptide attached to proinsulin could affect the refolding yield, because the leader peptide affects protein conformation and hydrophobicity. For example, a high refolding yield of 85% was achieved by one of the leader peptide’s high hydrophilic character.

There has been a report of using PFLC SEC column chromatography to refold the recombinant human proinsulin (Yuan et al. 2015). The propensity for protein aggregation is reduced by the gradual removal of the denaturant during buffer exchange while passing through a column (Batas and Chaudhuri 1996). Small molecules, such as urea and dithiotheritol (DTT), enter the pores of the resin and are separated from the unfolded protein molecules. The protein will start to fold into compact and partially folded conformation that can enter the resin pores as the chaotrope concentration around the unfolded species is reduced. Compared to PFLC, the dilution method of refolding proinsulin is more straightforward as it does not require the use of expensive membrane or chromatography column. The main disadvantage of using dilution method is a high buffer requirement especially for large-scale operation. PFLC-based protein refolding process may be more efficient as the protein is being refolded continuously with high productivity and low consumption of the refolding buffer.

There is no universal method to achieve high refolding efficiency and yield. It is important to optimize the refolding buffer compositions, protein concentrations, and conditions for each product individually, so as to assure the most economical and proper refolding process.

### Enzymatic cleavage

Insulin is synthesized in the human body as a single-chain precursor proinsulin (preproinsulin) consisting of chain A-, B-, and C-peptide (Mollerup and Frederiksen 2016). The formation of mature insulin requires the removal of C peptide by cleavage of Arg–Arg and Lys–Arg, leaving chain A and B connected by disulfide linkages.

Similarly, in “proinsulin” route of insulin production, the recombinant precursor human insulin/analogue molecules have to be enzymatically cleaved to remove the C-peptide to produce human insulin/analogue heterodimers. In insulin production, enzymatic cleavage most frequently consists of using two enzymes, trypsin and carboxypeptidase B (CPB). Trypsin is a serine endopeptidase which cleaves at the carboxy terminal of internal arginine and lysine residues (Harrison et al. 2015). The exopeptidase carboxypeptidase B (CPB) removes basic amino acids, such as arginine, lysine, and histidine, on the C-end as a result from trypsin activity (Balcerek et al. 2018). Table 14 presents a list of optimized conditions reported for enzymatic conversion of proinsulin to produce human insulin/analogues. A wide range has been reported for the mass ratio of proinsulin to enzyme, reaction temperature and duration. Here, we see that there is no fixed protocol to follow and optimization is required for every insulin purification scheme.

**Table 14:** Reported optimized conditions for enzymatic conversion of proinsulin (PI) to human insulin/analogue

Protein	Cleavage yield (%)	Cleavage duration	Trypsin	CPB	pH	Temp	References
rhPI	95	4 h	1 mg/L	4 mg/L	NA	30 °C	Petrides et al. (1995)
rhPI	90	1 h (in the presence of 10 mM hydrogen peroxide)	4.5 units of trypsin in 0.5 g/L human PI	20 units of CPB in 0.5 g/L human PI	7.5	25 °C	Son et al. (2008)
rhPI	77.7	16 h	1 mg/L trypsin in 0.5 g/L human PI	0.3 mg/L CPB in 0.5 g/L human PI	8.5	15 °C	Son et al. (2009)
rhPI	NA	30 min	Trypsin/substrate ratio 1:100	CPB/substrate ratio 1:400	7.5	37 °C	Bai et al. (2003)
rhPI	NA	16–18 h for each separate reaction with trypsin and CPB	A280 nm × volume of solution (dm^3^)/75	10 μl for every 150 AU of total protein determined in the main fraction	8.8	RTP	Zieliński et al. (2019)
rhPI or analogues	NA	1 h	Trypsin/substrate ratio 1:400	CPB/substrate ratio 1:2000	7.6	37 °C	Balcerek et al. (2018)
Five fused PIs	NA	3 h	0.45 unit/mg protein	0.2 unit/mg protein	7.5	15 °C	Min et al. (2011)
Interleukin-2-proinsulin	> 90	5 h	Enzyme/substrate ratio (1:600 w/w)	–	9.0	37 °C	Castellanos-Serra et al. (1996)
Novel PI	NA	NA	2000:1 mass ratio of protein to trypsin	1:1000 ratio of protein to CPB	NA	NA	Zimmerman and Stokel (2010)
N-terminal His_8_–Arg-tag	NA	30 min	PI-to-trypsin ratio (300:1)	PI-to-CPB ratio (600:1)	7.5	Ambient	Winter et al. (2002b)
Peptide fusion glargine	NA	5 h	9 units/mg protein	–	8.5	25 °C	Hwang et al. (2016)
Purified preproinsulin (98% purity)	NA	16 h	0.9 unit/mg protein	0.4 unit/mg protein	8.5	15 °C	Kim et al. (2015)
Renatured PI	NA	1 h	35 µg of trypsin per 1 mL of renatured sample (8 mg/ml)	0.6 µg of CPB per 1 mL of renatured sample (8 mg/ml)	7.5	37 °C	Astolfi et al. (2004)
Renatured PI	NA	5 h (trypsin) 30 min (CPB)	NA for trypsin concentration	0.075 U/mg arginine–insulin	7.5	14 °C (trypsin) 37 °C (CPB)	Redwan et al. (2007)
ZZ-R-PI	44	30 min	ZZ-R-PI/ trypsin ratio (1000:1 w/w)	ZZ-R-PI/ CPB ratio (2000:1 w/w)	8.0	NA	Nilsson et al. (1996)

*NA* information not available, *rhPI* recombinant human proinsulin, *CPB* carboxypeptidase B

The leader peptide and C-peptide can be concomitantly cleaved off by trypsin if arginine and lysine linkers are designed to attach the leader peptide to proinsulin (Min et al. 2011). The enzyme reaction rate can be influenced by the type of leader peptide linker that is connected to B chain. It has been found that the enzyme reaction rate is almost doubled when an arginine, instead of a lysine linker, is used (Min et al. 2011). Other than that, the hydrophobicity and length of the leader peptide can affect the tertiary structure of fused proinsulin, which can have an impact on the rate of enzymatic reaction (Min et al. 2011). The enzymatic reaction becomes quicker when there is more exposure of the cleavage site. Thus, it is important to evaluate the rate of enzymatic reaction and conversion yield when designing fusion partners for proinsulin, as the structure of the proinsulin molecule can have an effect on the rate of conversion.

The enzymatic digests introduce impurities, such as A21 desamido insulin, B30 des-threonine insulin, insulin ethyl ester, and arginine- and diarginine-insulin (Balcerek et al. 2018; Coleman et al. 2019). The most significant ones among these contaminants are A21 desamido insulin and B30 des-threonine insulin. The reaction temperature and the ratio of trypsin to CPB are both significant factors to monitor so as to reduce the formation of B30 des-threonine insulin (Min et al. 2011). Modification of trypsin/substrate ratio was found to result in significant changes in HPLC profiles of the digest products (Castellanos-Serra et al. 1996). Fluctuations in temperature between 4 and 37 °C have a strong influence on the rate of enzymatic digestion, with only a minimal effect on the proportion between human insulin and des-octa-insulin generated.

It is also important to monitor the entire length of enzymatic reaction, and to quench it at the maximal insulin point, because the B30 des-threonine insulin amount could increase with time (Min et al. 2011). The reaction time was molecule-dependent. It was found that the C-peptide was completely removed after 10 min of trypsinization, but the N-terminal extension of interleukin-2-proinsulin was completely removed after prolonged reaction of 5 h (Castellanos-Serra et al. 1996). The digestion can be monitored by RP-HPLC to determine the end point of reaction. There are a few methods to quench the enzymatic reactions. As the tryptic conversion of proinsulin to insulin is performed at around pH 7.5, the first method is to titrate down the pH of the reaction mixture to pH 2.5–3.5 by adding glacial acetic acid (Zimmerman and Stokell 2010) or trifluoroacetic acid (Nilsson et al. 1996). This is a suitable pH for directly loading the digest pool unto CEX, as the digest pool is at a pH lower than the pI of the properly folded insulin/insulin analogue. The second method involves the addition of a protease inhibitor, such as aprotinin, to the digest pool to stop the enzymatic digestion prior to chromatographic purification (Watson et al. 2018). This method is useful if the next column after digestion is an AEX/MMA, because there is no need to titrate down the pH. There has also been a report of using isopropanol (final concentration: 40%) to stop the cleavage (Bai et al. 2003).

Prior to proteolysis reaction, citraconylation can be performed to regulate enzymatic digestion and to avoid insulin degradation (Mikiewicz et al. 2017; Zieliński et al. 2019). The substrate-binding site of bovine trypsin has a negatively charged Asp-189 residue, which can have ionic interactions with the substrate through their positively charged amino acid side chains (Evnin et al. 1990; Gráf et al. 1988). The trypsin cleavage sites can be altered by the addition or deletion of positive charges, as trypsin only cleaves peptide bonds behind positively charged residues. In citraconylation, the insulin precursor is treated with citraconic or maleic acid anhydride, in which the lysine residue (LysB29) is blocked by acetylation reaction. As a result, its positive charge is converted into negative charge and thus prevents the hydrolysis of the peptide bond from C-end of lysine in B chain. The blocking of lysine and arginine residues by citraconic anhydride is reversible to allow for a controlled tryptic digestion (Zieliński et al. 2019). Citraconic anhydride blocks primary amine groups at alkaline pH values above 8. At an acidic pH under pH 4, the amide linkage is hydrolyzed and citraconic acid is released to yield the original amine (Zieliński et al. 2019).

A typical citraconylation protocol in insulin production entails incubation of the solution in pH 8.4–9.3 with stirring at 25 °C for 2–2.5 h (Balcerek et al. 2018; Hwang et al. 2016; Zieliński et al. 2019). After precursor proteolysis, decitraconylation is carried out by incubation of proinsulin in pH 2.5–3.0 in the cold or room temperature for several hours (Balcerek et al. 2018; Hwang et al. 2016; Son et al. 2009; Zieliński et al. 2019).

The correctness of citraconylation and trypsinization reactions can be verified by mass spectrometry (Zieliński et al. 2019). Citraconylation is able to reduce the amount of several types of digest contaminants. As the presence of B30 des-threonine insulin contaminant is a result of B30 threonine elimination by trypsin, citraconylation was able to reduce the amount of this contaminant by 5–10% (Balcerek et al. 2018). Citraconylation of lysine residues in human proinsulin blocked trypsin cleavage, reduced the formation of des-threonine insulin from 13.5 to 1.0%, and hence increased the production yield of active insulin (Son et al. 2009). Citraconylation minimized the formation of Arg (B31)-insulin and raised the enzymatic conversion yield of glargine insulin by 3.2-fold, as compared to without citraconylation (Hwang et al. 2016).

To eliminate or reduce certain unwanted cleavage by-products, enzymes such as enterokinase, Asp-N endoproteinase, and Kex2 protease have been explored in insulin purification (Balcerek et al. 2018). In particular, Kex2 protease has a high specificity and good proteolytic stability even during extended incubation, in which this is an advantage over commonly used enzymes (Balcerek et al. 2018). Due to the specificity of enzyme action, it may be necessary to introduce modifications in the proinsulin peptide sequence to direct cleavage at the desired site if there is no naturally occurring cleavage site recognizable by the enzyme (Balcerek et al. 2018). For example, after switching the lysine residue with an alanine residue at position 64 of the native proinsulin sequence, the theoretical enzyme reaction yield increased, because the formation of arg-AO-insulin during the trypsin cleavage was prevented (Zimmerman and Stokell 2010).

The conditions adopted in enzymatic conversion can have an effect on the quantity and variety of contaminants generated. It is important to optimize the cleavage step to increase the yield of properly cleaved human insulin/analogue and to reduce the amount of undesirable digest by-products. A higher purity achieved in the digest pool will simplify subsequent purification steps, thus leading to greater cost savings in production.

### Formulation

After final chromatographic purification, the product must be conditioned for subsequent formulation into a drug product that is stable during storage. The main risk factors involved when handling the product after the final chromatographic purification are deamidation of the insulin molecule and formation of aggregates/fibrils (Brange 1994). Aggregation can occur after stressing the molecule by agitation, low pH, high temperature, high protein concentration, high ionic strength, and in the presence of denaturants or high concentration of organic solvent (Nielsen et al. 2001; Tiiman et al. 2013). Deamidation can occur when there is prolonged exposure to acidic conditions to form A21 desamido insulin (Mollerup et al. 2010). Thus, to prevent insulin fibrillation and deamidation, residual salts and buffers from chromatographic purification must be removed via crystallization and lyophilisation. The insulin crystals can be isolated by centrifugation, decantation or filtration, and eventually, they are washed to remove the residual zinc content. After zinc crystallization, the next step that follows is freeze-drying (Coleman et al. 2019). The recombinant product will be solubilized when ready for packaging (Zimmerman and Stokell 2010).

Specific additives can be added to the formulation to prevent bacterial growth and protein aggregation, or to modify the in vivo absorption kinetics of insulin. For example, m-cresol could be added to suppress insulin amyloid formation (Ohno et al. 2019). Complexation of insulin with zinc and/or protamine can generate intermediate- or long-acting formulations (Waller and Sampson 2017).

## Conclusions

Insulin is a scarce commodity, with global demands projected to rise due to increasing prevalence of diabetes. The chronic nature of diabetes means that patients require long-term insulin treatment, and this can place heavy financial burden on the healthcare systems and individuals. It is therefore imperative that all the manufacturers and stakeholders continue to play their parts in making further progresses in therapeutic recombinant insulin development and production. A comprehensive review of recombinant human insulin and its analogues’ downstream processing via the “proinsulin route” from *E. coli*-based system has been presented. We have provided pertinent examples of critical production stages and discussed the practical aspects of integrating every procedure into a multimodal purification scheme. As evidenced from this review, a large number of purification and conversion steps are required to recover and purify the recombinant insulin product. This review may serve as a guide to help downstream process scientists develop a more efficient and economical process for the production of human insulin and its analogues.

## Data Availability

All data generated or analyzed during this study are included in this published article.

## Abbreviations

AC: Affinity chromatography
ACN: Acetonitrile
AEX: Anion exchange chromatography
BME: β-Mercaptoethanol
CEX: Cation exchange chromatography
CPB: Carboxypeptidase B
CNBr: Cyanogen bromide
Cryst.: Crystallization
CV: Column volume
DTT: Dithiothreitol
ELISA: Enzyme-linked immunosorbent assay
fMet: *N*-Formylmethionine
GdnHCl: Guanidine hydrochloride
GSH: Reduced glutathione
GSSG: Oxidized glutathione
HIC: Hydrophobic interaction chromatography
HPLC: High-performance liquid chromatography
IB: Inclusion body
IEX: Ion-exchange chromatography
IMAC: Immobilized metal ion affinity chromatography
MMC: Mixed-mode chromatography
NA: Information not available
PEG: Polyethyleneglycol
PFLC: Protein-folding liquid chromatography
pH-PPT: pH precipitation
PI: Proinsulin
pI: Isoelectric point
rhPI: Recombinant human proinsulin
RP: Reversed-phase chromatography
RTP: Room temperature
SDS-PAGE: Sodium dodecyl sulfate-polyacrylamide gel electrophoresis
SEC: Size exclusion chromatography
SMB: Simulated moving bed
TFA: Trifluoroacetic acid
ZnCl_2_-PPT: Zinc chloride precipitation
